# Supplementary material for: Proteomic Signatures of Corals from Thermodynamic Reefs
Source: Microorganisms. 2020 Aug 1;8(8):1171. doi: 10.3390/microorganisms8081171 (PMC7465421; doi:10.3390/microorganisms8081171)
Supplement: Supplementary file 1 [file microorganisms-08-01171-s001.zip › Mayfield Microorganisms 2020 supplemental material.pdf]

Appendix

1. *Online supplemental methods*

1.1. Protein quantification & quality assessment

Upon dissolving the proteins in triethylammonium bicarbonate (TEAB) with 0.067% (final concentration) sodium dodecyl sulfate (SDS) via vigorous vortexing (Vortex Genie, USA), a 5-µL aliquot was diluted 10-fold in water and quantified using a bicinchoninic acid (BCA) assay from Pierce (USA) according to the manufacturer's recommendations. It should be noted that this dilution step was critical since both TEAB and SDS can interfere with the BCA assay at higher concentrations. A second, 1-2-µL aliquot of protein was mixed with 2X Laemmli sample buffer [60] (BioRad, USA, cat. 161-0737), boiled at 95% for 5 min, and loaded into a PhastGel™ 4-15 gradient (isoelectric point) polyacrylamide gel from GE Healthcare (USA, cat. 17-0678-01). The gel was then loaded into the Phast System™ (GE Healthcare) after inserting two PhastGel SDS buffer strips (cat. 17-0516-01). Proteins (1-3 µL) were run alongside 1 µg/µL of BSA standard (0.2-0.3 µL) and 1 µL of SeeBlue™ Plus2 Pre-stained protein standard (Thermo-Fisher, Scientific [TFS] cat. LC5925) under separation method 3. After ~2 hr, the gel was washed thrice with water and then stained with 10-20 mL of SimplyBlue™ Safe Stain (Invitrogen, USA) for 1 hr at room temperature (RT). The stained gel was then washed repeatedly with water until bands could be visualized with the naked eye (typically overnight).

After quantifying proteins (BCA) and ensuring that they were not degraded (PhastGels), the remaining proteins were dried and resuspended in TEAB-SDS as described in the main text. To the 12 coral samples and 2 normalizers (described in the main text & **Table 2**), 1 µL of tris-2-carboxyethyl-phosphine (TCEP; Sigma-Aldrich, USA) was added to reduce the dissolved proteins' disulfide bonds. Samples (n=14) were then vortexed, centrifuged at 15,000 RPM for 5 min (hereafter simply referred to as "spun"), and incubated at 60ºC for 1 hr. Samples were re-spun and alkylated with 1 µL of freshly prepared 84 mM iodoacetamide (Sigma-Aldrich) in water, vortexed, spun, and incubated in the dark at RT for 30 min. Samples were once again spun and then mixed with 10 µL of 0.1 µg/µL sequencing grade modified trypsin (Promega, USA; cat. V5111) for 3 hr at 37ºC. Then, an additional 1 µL of trypsin was added, and proteins were digested overnight at 37ºC. After spinning, samples (~43 µL) were dried in a Labconco speed-vac (USA) and resuspended in 30 µL of 0.5 M TEAB (without SDS). They were then mixed with 50 µL of isopropanol and 17-22 µL of the appropriate iTRAQ reagent (SCIEX iTRAQ Reagent-8plex 25 U kit, USA) according to the manufacturer's recommendations (lot#A7012; see **Table 2** for reagent volumes for each sample.). With the exception of label 113, which was dedicated to the two normalizers, the remaining labels were assigned randomly to avoid running all samples from any particular temperature treatment or site of origin in the same batch (**Table 2**).

Samples were vortexed, spun, labeled at RT for 2 hr, quenched with 100 µL of distilled water for 30 min, and dried to 10-20 µL in the speed-vac. Next, samples from each batch of 7 (the normalizer [113] plus the remaining 6 target samples for batches A & B; **Table 2**) were combined into the same tube and dried to completion in the speed-vac. The two pellets (105 µg of protein each) were washed thrice with water, drying to completion after each wash. After the final round of drying, samples were resuspended in 20 µL of 2% acetonitrile with 0.1% formic acid prior to separation through an Acclaim™ PepMap™ (75 µm×15 cm), nanoViper column (TFS cat. 164568) followed by nano-liquid chromatography (LC) on an Easy Nano LC™ 1000

(TFS) featuring a Nanospray Flex ion source (TFS) as described previously [61]. Peptide eluates from a 2-98% acetonitrile gradient were individually run on a Q Exactive™ Orbitrap mass spectrometer (MS; Fourier transform) in MS2 mode with high-energy collisional dissociation (HCD) activation (28 eV) as described previously [61], with a scan range of 150-1600 m/z.

1.2. Proteomic data analysis

RAW data files from the MS were loaded into Proteome Discoverer® ver. 2.2 (TFS), and, in most cases (see exceptions below.), the default conditions were used to query the *Seriatopora hystrix* holobiont transcriptome [31] (described in the main text). A peak integration tolerance of 20 ppm was set, and the peak integration method was based on the most confident centroid algorithm. Precursor and fragment mass tolerances were 10 ppm and 0.02 Da, respectively, and up to two missed cleavages were permitted. The raw data (as RAW, MZML, and MZID files) have been deposited on the University of California San Diego's (USA) MassIVE data repository (massive.ucsd.edu; accession: MSV000085863), where they can be re-analyzed using MassIVE's open-access MS software. MassIVE is a full member of the Proteome Xchange Consortium (http://www.proteomexchange.org/), and the dataset has been cross-referenced in the Proteome Xchange repository under accession PXD020679. The same dataset was also published on NOAA's National Centers for Environmental information (NCEI) database (https://www.ncei.noaa.gov/) under accession 0216077 (https://data.nodc.noaa.gov/cgi-bin/iso?id=gov.noaa.nodc:0216077) and will later be cross-referenced in NOAA's Coral Reef Information System (CoRIS; https://www.coris.noaa.gov/) under the NCEI accession number. All data from the MS have been distilled into an Excel spreadsheet (online supplemental data file [**OSDF**]) that has been posted on the *Microorganisms* website.

Unlike for transcriptomic and genetic analyses, in which contigs can be mapped to individual Symbiodiniaceae lineages, tryptic peptides are generally too short (6-10 amino acids [AA]) to do so with confidence. Indeed, in many cases herein, sequenced peptides could not be confidently ascribed even to host or endosymbiont. For these reasons, sequences were not assigned to exact Symbiodiniaceae lineages. Unlike BLAST, MS algorithms do not query protein sequences; instead, MS peaks are used to infer AA molecular weights. For highly conserved proteins, the software is unlikely to assign large numbers of peptides to the correct compartment of origin with statistical confidence. Such confidence is derived from interpretation and statistical analysis of peptide score *q*-values, which are similar to the *e*-values used in nucleic acid searches but instead corrected against decoy databases such that false discovery rates (FDR) can be calculated (discussed in detail below).

Because of the concern with inadvertently assigning sequenced peptides to the incorrect compartment of origin, an additional rule that two peptides (each >6 AA) mapped to the same protein was enacted. It is possible that increasing the mapping stringency even further (*e.g.*, 3-4 peptides mapping to the same conceptually translated protein) could ultimately lead to an uncovering of the exact Symbiodiniaceae lineage from which the protein emerged, though this would result in such a low number of proteins (<5-10) that key facets of the molecular biology hoped to be elucidated by this proteomics approach would be missed. Furthermore, as discussed in the main text, an alternative, qPCR-based approach revealed that our corals predominantly hosted exclusively *Cladocopium* spp. endosymbionts (**Table 1** & references therein). Whether the majority of the Symbiodiniaceae proteins sequenced and characterized were also from *Cladocopium*, as opposed to other, background endosymbiont types, remains to be determined.

In addition to the minimum peptide length of 6 AA, 144 AA was set as the maximum. For fasta library querying, decoy and contaminant databases were searched simultaneously such that FDRs could be calculated. Only proteins whose confidence scores fell below the FDR-adjusted *p*-value of 0.01 were considered. Of these proteins, only those with an iTRAQ label were included. Unlike DNA sequencing, in which only DNAs/cDNAs with tags are sequenced, the MS generates a mix of peptide sequences with and without the iTRAQ tags, allowing for the estimation of labeling efficiency (typically 10-30%; see Discussion.). It is worth mentioning that the remaining, untagged proteins could be used for future presence/absence analyses.

As an additional quality control criterion, it was established *a priori* that only proteins sequenced in both iTRAQ batches would be considered. This is because, despite having 1) randomly allocated corals from the four site of origin x temperature treatment interaction groups to each of the two iTRAQ batches and 2) run the identical, normalizer sample in both batches, it was nevertheless possible that batch effects could have led to type I statistical errors. For instance, if a peptide was only sequenced in batch A but not in batch B, concentrations of 0 were *not* assigned to this protein in samples of the latter batch; it was instead omitted entirely. Of the high-confidence proteins found in each batch with iTRAQ labels (**Figure S1b**), it was required that two mapped to the same conceptually translated contig so that greater confidence in the protein identity and compartment of origin would be obtained (discussed above).

1.3. Proteomic statistical analysis-multivariate

When dealing with large numbers of molecules (*e.g.*, 'OMICs datasets) in which the number of cellular targets is far greater than the number of samples, many inferential multivariate approaches cannot be used, namely multivariate ANOVA (MANOVA). However, exploratory-based multivariate approaches, such as principal components analysis (PCA) and multi-dimensional scaling (MDS) *can* instead depict multivariate differences between samples in a semi-quantitative manner, and PCA on correlations and MDS of standardized data were carried out herein to depict relationships and similarity among samples, respectively. PCA and MDS were performed as described in the main text with 1) all 30 proteins (including 4 microbial proteins & 3 proteins that could not be confidently assigned a compartment of origin), 2) the 11 host proteins alone, and 3) the 12 endosymbiont proteins alone. As part of the PCA, k-means clusters were defined by JMP® Pro (ver. 14.2), and those samples that clustered with the incorrect site of origin have been highlighted in the corresponding figures. In contrast, MDS ellipses (**Figure 2**) were drawn by eye. Unlike MANOVA, similarity-based permutational MANOVA (PERMANOVA) can be used to uncover multivariate mean differences when the number of targets is larger than the number of samples. Therefore, PRIMER (ver. 6., UK) was used to carry out PERMANOVA of temperature regime, site of origin, and their interaction on the 30-protein dataset (all protein data were first standardized such that highly concentrated and low-abundance proteins were given equal weight.). An alpha level of 0.05 was set for PERMANOVA (unrestricted permutation of raw data [type III model]) using a Bray-Curtis similarity matrix as the input data format.

1.4. Differentially concentrated proteins (DCPs)

A response screening analysis (RSA) of temperature treatment, site of origin, and their interaction was carried out with JMP Pro; this is an FDR-controlled comparison that searches for proteins whose concentrations are most affected by experimental factors in a manner that limits the possibility of generating type I statistical errors when performing numerous comparisons.

Proteins whose FDRlogworth values were >2 (equivalent to an FDR-adjusted *p*-value<0.01) and for which no tank effect (nested within temperature x site of origin) was documented were considered to be DCPs. It is worth noting here, that, in contrast to the qPCR-derived mRNA expression data (**Table 1** & references therein), protein concentrations (as ratios to the normalizer sample) were *not* further normalized to a host/endosymbiont protein ratio since the relative proportion of host vs. dinoflagellate did not vary across the 12 proteomic samples (*i.e.*, no bleaching was documented,); however, there was a site effect on endosymbiont density (see main text Results.). To corroborate this, the average endosymbiont protein concentration was calculated for each of the 12 samples and compared in a one-way ANOVA; the Symbiodiniaceae protein signal was statistically similar across samples (F=1.42, p=0.171), signifying that no sample was significantly enriched with dinoflagellate proteins relative to others. Furthermore, there were no effects of temperature, site of origin, or their interaction on the average Symbiodiniaceae protein concentration (all p>0.05); this is further evidence that similar amounts of dinoflagellate protein (relative to host coral protein) were extracted from each sample. In contrast, concentrations of individual Symbiodiniaceae proteins varied in response to experimental factors; such differences are discussed in the main text.

1.5. Proteomic predictive modeling (PPM)

In addition to attempting to uncover proteins involved in the variable temperature response, the second analytical goal of this work was to identify proteins, or suites of proteins (hereafter termed "proteins of interest" [POIs]) that could be used to develop models that would enable the future predicting of coral behavior *in situ*. Please see the main text for a more formal differentiation between POIs and DCPs. Two PPM were tested. First, stepwise discriminant analysis (SDA) was used to identify the proteins that best led to separation of samples from the individual experimental factors such that the most parsimonious model that correctly classified 100% of samples was generated. The confidence was determined by "burning in" random samples as the training data, using the remainder as validation samples (typically 70/30% training/validation). An artificial intelligence (AI)-based algorithm from JMP Pro then simulated this random sample training vs. validation step several million times until the most parsimonious model was developed (*i.e.*, the fewest number of proteins resulting in the highest level of statistical confidence). The AI then guessed which sample was from which treatment, and the guess was compared to the actual sample identity in a double-blind manner to determine correctness (reported as misclassification rates in the main text).

Stepwise regression analysis (SRA) was also used to build protein-based models for the coral response to upwelling using a binomial (for temperature regime and site of origin) or multinomial (temperature x site of origin interaction) model, with the 30 proteins as predictors. A forward-built, AI-driven, minimum-Bayesian information criterion modeling platform was utilized on JMP Pro. In most cases, the DCPs were also those most likely to feature in the predictive models (**Figure S1e**); for instance, if a protein was differentially affected by temperature, it was almost certainly in the SDA and SRA temperature models (& vice versa).

2. *Supplemental table*

Table S1. Permutational MANOVA. PERMANOVA of the effects of site of origin, temperature treatment, and their interaction on the *Seriatopora hystrix*-Symbiodiniaceae partial proteome (n=30 proteins). The PERMANOVA model featured unrestricted permutation of raw

data, and data were standardized prior to building the Bray-Curtis similarity matrix (which was also used for multi-dimensional scaling; see **Figure 2**.). SS=sum of squares.

**Source of variation**	**df**	**MS**	**Pseudo-*F***	***p***
Site of origin	1	48.4	2.023	0.064
Temperature treatment	1	34.6	1.45	0.182
Site x temperature	1	26.9	1.12	0.337
Residual (SS=168)	8	23.9		

3. Supplemental figures

Figure S1. Venn diagrams. Venn diagrams depicting iTRAQ batch effects for all sequenced proteins (a) and those with iTRAQ labels only (b). Venn diagrams demonstrating overlap between the iTRAQ-based proteomic approach taken herein (all proteins [c] and labeled proteins only [d]) with the 2-dimensional gel electrophoresis (2DGE) strategy applied previously [31]. No protein was found to be differentially concentrated by both methods, and only 1 of the 30 proteins that passed all quality control criteria herein (**Figure 1**) was identified previously [31]: c62707_g1 (a host coral beta-gamma crystallin found by 2DGE to be down-regulated under variable temperature exposure). A comparison of "proteins of interest" (POIs; including differentially concentrated proteins [DCPs]) identified by three statistical approaches (n=4 DCPs+7 POIs): response screening analysis, stepwise discriminant analysis, and stepwise regression (e). Select proteins have been labeled.

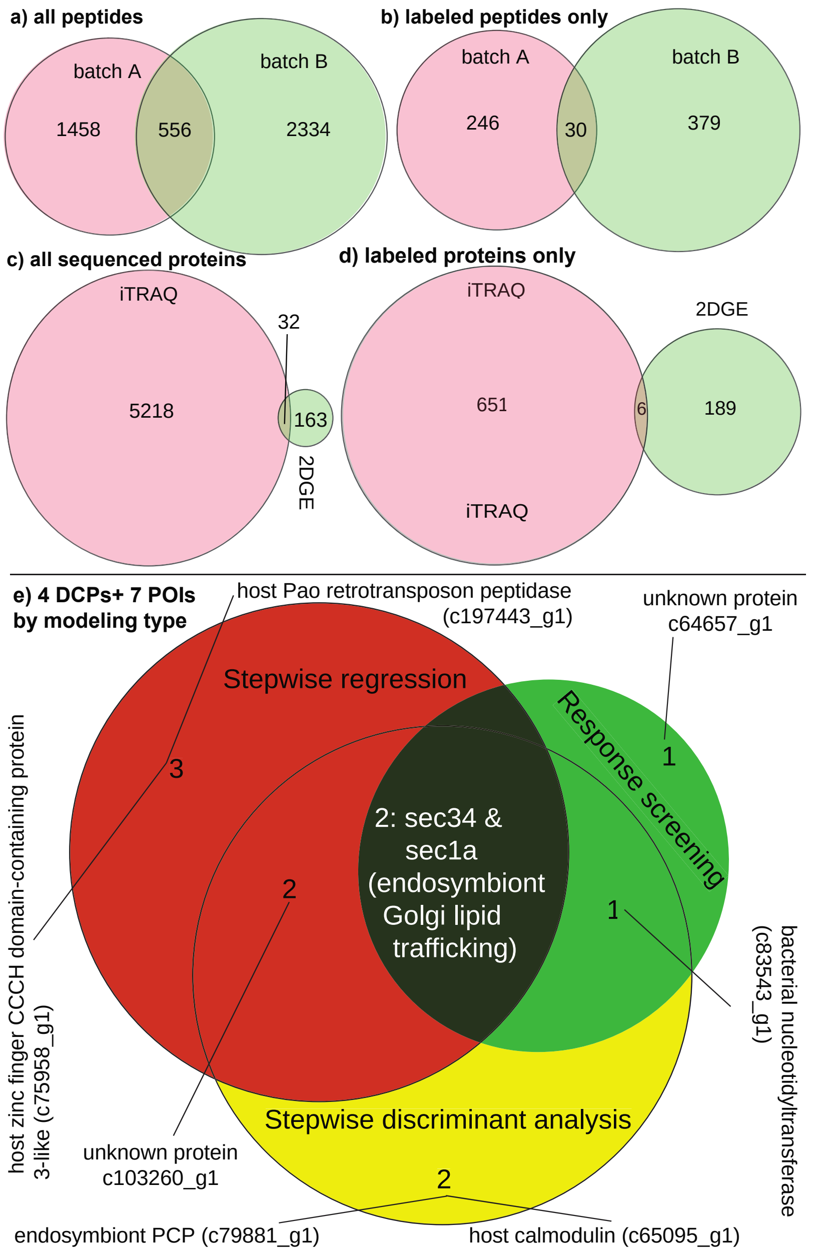

Figure S2. t-distributed stochastic neighbor embedding (t-SNE) analysis of the 95,000-contig *Seriatopora hystrix*-Symbiodiniaceae transcriptome. Despite the lack of correlation between gene expression and protein concentrations discussed in the main text, both proteomic (**Figure 2**) and transcriptomic approaches [31] were generally able to distinguish corals by site of origin. The t-SNE was carried out at a perplexity of three (300 iterations) with an R-interfaced add-in run on JMP Pro via JMP scripting language (JSL). S=stable temperature regime. V=variable temperature regime.

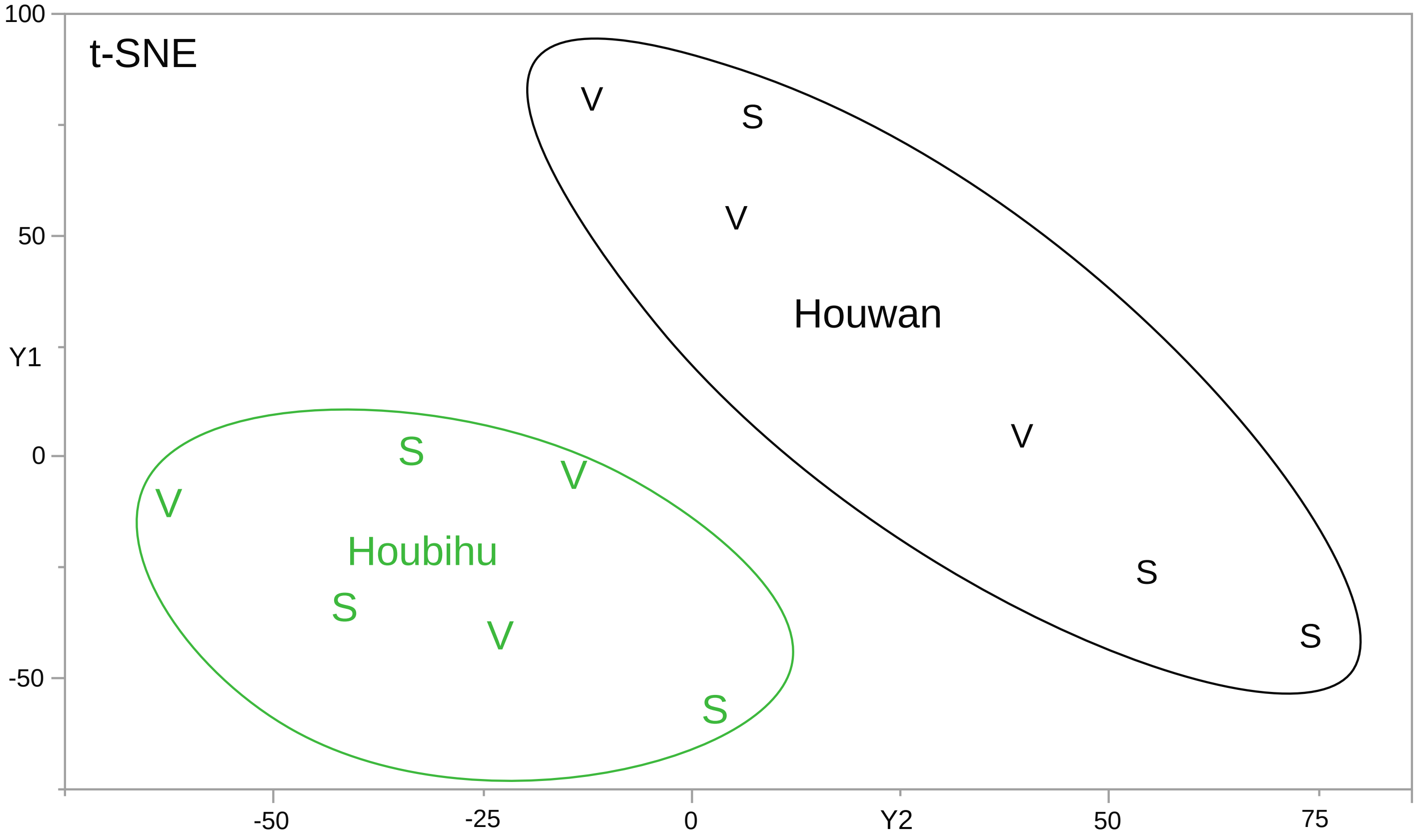

4. Supplemental references

31. Mayfield, A.B.; Wang, Y.B.; Chen, C.S.; Chen, S.H.; Lin, C.Y. Dual-compartmental transcriptomic+proteomic analysis of a marine endosymbiosis exposed to environmental change. *Mol. Ecol*. **2016**, *25*, 5944-5958.
60. Laemmli, U.K. Cleavage of structural proteins during the assembly of the head of bacteriophage T4. *Nature* **1970**, *227*, 680-685.
61. Desoubeaux, G.; Chauvin, D.; Piqueras, M.C.; Bronson, E.; Bhattacharya, S.K.; Sirpenski, G.; Bailly, E.; Cray, C. Translational proteomic study to address host protein changes during aspergillosis. *PLoS ONE* **2018**, *e0200843*.
